# Highly Integrated Multi‐Material Fibers for Soft Robotics

**DOI:** 10.1002/advs.202204016

**Published:** 2022-11-22

**Authors:** Andreas Leber, Chaoqun Dong, Stella Laperrousaz, Hritwick Banerjee, Mohamed E. M. K. Abdelaziz, Nicola Bartolomei, Bastien Schyrr, Burak Temelkuran, Fabien Sorin

**Affiliations:** ^1^ Institute of Materials École Polytechnique Fédérale de Lausanne Lausanne 1015 Switzerland; ^2^ The Hamlyn Centre for Robotic Surgery Imperial College London London SW7 2AZ UK; ^3^ Department of Metabolism Digestion and Reproduction Faculty of Medicine Imperial College London London SW7 2AZ UK

**Keywords:** multi‐material fibers, sensing and actuation, soft robotics, steerable catheters and endoscopes, thermal drawing

## Abstract

Soft robots are envisioned as the next generation of safe biomedical devices in minimally invasive procedures. Yet, the difficulty of processing soft materials currently limits the size, aspect‐ratio, manufacturing throughput, as well as, the design complexity and hence capabilities of soft robots. Multi‐material thermal drawing is introduced as a material and processing platform to create soft robotic fibers imparted with multiple actuations and sensing modalities. Several thermoplastic and elastomeric material options for the fibers are presented, which all exhibit the rheological processing attributes for thermal drawing but varying mechanical properties, resulting in adaptable actuation performance. Moreover, numerous different fiber designs with intricate internal architectures, outer diameters of 700 µm, aspect ratios of 10^3^, and a fabrication at a scale of 10s of meters of length are demonstrated. A modular tendon‐driven mechanism enables 3‐dimensional (3D) motion, and embedded optical guides, electrical wires, and microfluidic channels give rise to multifunctionality. The fibers can perceive and autonomously adapt to their environments, as well as, probe electrical properties, and deliver fluids and mechanical tools to spatially distributed targets.

## Graphical abstract

The preform‐to‐fiber thermal drawing of thermoplastic elastomers enables the fabrication of soft robots with extreme aspect ratios and intricate internal multi‐material structures, resulting in diverse functionalities. The soft robotic fibers can perceive their environments and autonomously adapt to changes thereof, as well as, probe electrical properties, and deliver fluids and mechanical tools to spatially distributed targets.

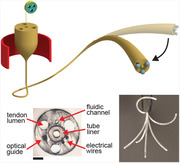

## 1 Introduction

Soft robots hold great potential in applications involving the human body because they can safely conform and interact with fragile and dynamic environments.^[^ ^1^ ^]^ In such compliant and deformable robotic systems, movement is enacted by one of numerous possible actuation mechanisms, based on tendons,^[^ ^2^ ^]^ pneumatics,^[^ ^3^ ^]^ hydraulics,^[^ ^4^ ^]^ shape memory effects,^[^ ^5^ ^]^ or electrical^[^ ^6^ ^]^ or magnetic^[^ ^7^ ^]^ activation—each with their own strengths and weaknesses.^[^ ^8^, ^9^, ^10^, ^11^ ^]^ To accomplish meaningful tasks, actuation is often coupled with additional functionalities, such as sensing,^[^ ^12^, ^13^ ^]^ imaging,^[^ ^14^ ^]^ optical,^[^ ^15^ ^]^ or electrical^[^ ^16^ ^]^ transmission, and drug delivery.^[^ ^17^ ^]^

Multifunctional soft robots are particularly promising in tubular form to act as steerable endoscopes and catheters in minimally invasive procedures.^[^ ^18^, ^19^, ^20^ ^]^ However, creating the soft multi‐material assemblies, which are necessary for dexterous movement and embedded functionality,^[^ ^9^, ^11^ ^]^ at relevant diameters (<3 mm) and lengths (>300 mm) is a feat that is highly challenging, for both conventional extrusion‐based techniques^[^ ^21^, ^22^, ^23^ ^]^ and approaches adopted from macroscopic soft robotics relying on molding,^[^ ^24^, ^25^, ^26^, ^27^, ^28^ ^]^ stereolithography,^[^ ^29^ ^]^ 3D printing,^[^ ^30^ ^]^ and manual processing.^[^ ^31^ ^]^

We hypothesized that such high‐aspect‐ratio materials constructs could be realized by the thermal drawing process, which is conventionally employed for the fabrication of optical fibers and typically yields fibers of diameter 125 µm and length 1000 km.^[^ ^32^ ^]^ Beyond optical fibers, advancements in process technology have resulted in the scalable manufacturing of fibers incorporating intricate microstructures and a myriad of different materials.^[^ ^33^, ^34^, ^35^, ^36^, ^37^ ^]^ Recently, the breadth of compatible materials has been extended to soft thermoplastic elastomers,^[^ ^38^ ^]^ acting as an enabling technology for fiber‐based sensors^[^ ^39^, ^40^ ^]^ and energy harvesters.^[^ ^41^ ^]^ However, the potential of thermal drawing as an advanced materials and processing platform for soft robotics has thus far remained untapped.

In this article, we present novel materials combination, processes, and architecture designs to realize soft robotic fibers with diameters as thin as 700 µm that embed sensing capabilities and can accurately accomplish extensive 3D motion. Fabrication via the scalable thermal drawing technique enables direct incorporation of active elements in precise architectures within the thermoplastic‐elastomer‐clad fibers, including optical guides, metallic wires, microfluidic channels, and tool‐guiding liners, giving rise to a wide range of functionalities beyond mere actuation. The fibers can perceive their environments and autonomously adapt to changes thereof, as well as, probe electrical properties, and deliver fluids and mechanical tools to spatially distributed targets, thus promoting the fibers to nimble and capable soft robots.

## 2 Results and Discussion

**Figure 1:**
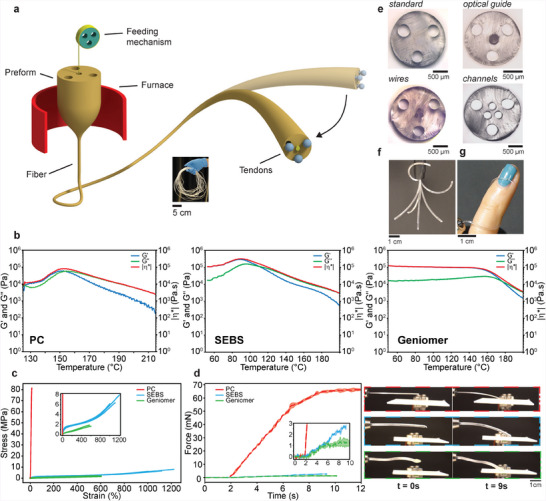
Soft robotic fibers fabrication, structure, and actuation. a) Schematic of the thermal drawing fabrication technique and the tendon‐driven actuation. The inset shows a photograph of a 15 m‐long continuous fiber. b) Oscillatory shear rheology tests showing the storage modulus (*G*’), loss modulus (*G*”), and complex viscosity (|η*|) as a function of temperature for the three material options PC, SEBS, and Geniomer. c) Stress–strain curves for PC, SEBS, and Geniomer. d) Blocking force experiment shown as the generated force applied onto a load cell by robotic fibers made of the three different materials PC, SEBS, and Geniomer (the solid line represents the mean and the shaded area represents standard deviation), as well as, photographs illustrating the experiment (from top to bottom: PC, SEBS, Geniomer). e) Optical micrographs of several produced fiber cross‐sections of SEBS. f) Merged sequence of photographs of a soft robotic fiber of SEBS, illustrating the range and complexity of motion. g) Photograph of a soft robotic fiber of SEBS of diameter 700 µm that wraps around a finger.

Our fabrication and actuation concept is illustrated in **Figure** **1**a. The thermal drawing process relies on the viscous flow of materials under heat and tension to convert a macroscopic materials assembly, termed preform, into a long fiber (Figure 1a inset).^[^ ^33^ ^]^ In the process, the cross‐sectional design is maintained from the preform to the fiber and is merely uniformly scaled down—at diameter ratios of 10 to 30 in this work. The diameter, which is continuously monitored, can be dynamically controlled by the feeding speed of the preform and the winding speed of the fiber. Prior to the thermal drawing step, complex geometrical features are readily realized at the level of the macroscopic preform using standard compression molding, such as holes that become microchannels in the fibers. Additionally, several materials can be combined during the assembly of the preform, which are subsequently co‐drawn and result in multi‐material fibers. By extending the thermal drawing process with a feeding mechanism, 1D elements, such as optical guides or metallic wires, can also be integrated in the fiber.^[^ ^42^ ^]^ Their placement within the fiber architecture is dictated by specifically arranged holes in the preform. During the process, the holes shrink alongside the whole cross‐sectional structure, allowing the surrounding material to latch on to the fed element and pulling it along. Practical considerations of the fabrication, including the preform manufacturing and thermal drawing step, are detailed in Section 4. The actuation of the fabricated fibers is achieved through a tendon‐based mechanism. It encompasses three tendons that are placed within lumens arranged at 120° within the fiber structure. Note that the tendons can be incorporated during thermal drawing using the feeding mechanism for large required fiber lengths, or inserted in the lumens in post‐processing for smaller sections. By fixing the tendons at the distal fiber end and concertedly pulling on them at the proximal end, the fibers bend in a controlled fashion with two degrees of freedom.

With the concept of the robotic fibers laid out, we address next the materials selection, which is focused, on one hand, on the rheological properties that drive the fabrication, and, on the other hand, on the mechanical properties that define the actuation performance. We also consider the ability to maintain microchannel integrity during the drawing process. We identified three polymers, which share similar rheological processing attributes but differ in their mechanical properties: Polycarbonate (PC), poly(styrene‐*b*‐(ethylene‐*co*‐butylene)‐*b*‐styrene) (SEBS), and Geniomer, a copolymer of aliphatic isocyanite and polydimethylsiloxane. In oscillatory shear rheology experiments (Figure 1b), all three materials display a behavior that is generally linked to compatibility with the thermal drawing process;^[^ ^38^ ^]^ specifically, a rapid decrease in storage modulus with temperature, in contrast to a slow decrease in loss modulus, resulting in a distinct cross‐over point. The temperature of the cross‐over is associated with the lower limit of the processing window and is 140 °C for PC, 110 °C for SEBS, and 176 °C for Geniomer. For all three materials, the complex viscosity remains at an elevated level in this temperature range (10^4^–10^5^ Pa s^−1^ for PC, 10^4^–10^5^ Pa s^−1^ for SEBS, and 10^3^–10^4^ Pa s^−1^ for Geniomer). A high viscosity is critical for thermal drawing, because it enables the material to sustain the large stresses during drawing and to maintain structural integrity. Indeed, micro‐scale geometrical features and textures can be preserved in the fibers, because a high viscosity slows down thermal reflow driven by surface tension.^[^ ^43^ ^]^

This is well exemplified by the preservation of the integrity of hollow channels within the fibers, destined to be used as micro‐fluidic conduits for sampling or delivery. Reflow driven by Laplace pressure during thermal drawing has been studied extensively in recent years.^[^ ^43^, ^44^ ^]^ The surface tension of typical elastomers such as SEBS being similar to the one of PC, of the order of 20–40 mN m^−1^,^[^ ^45^ ^]^ one can expect minimum reflow at viscosities of 10^3^–10^4^ Pa s^−1^ and typical feature sizes of 10–100 µm.^[^ ^43^ ^]^ The choice of block co‐polymer elastomers with relatively small surface tension and high viscosity during processing participate in the ability to fabricate fibers with microstructures such as hollow channels.

Next, we address the mechanical properties through tensile testing (Figure 1c). As expected for a thermoplastic, PC exhibits a linear stress–strain curve with a high modulus of 789 MPa and low elongation at break of 14%. In contrast, the two thermoplastic elastomers that display a non‐linear behavior are significantly softer and more deformable (for SEBS 100% modulus of 1.56 MPa and elongation at break of 1114%; for Geniomer 100% modulus of 0.48 MPa and elongation at break of 573%). We expect a soft and deformable material to be generally preferable because it enables a large range of bending angles at low activation forces. Additionally, the softness diminishes the risk of tissue damage in the targeted biomedical applications. However, the mechanical properties also control the output force that can be generated by the actuator. We investigated the effect of material on the actuation performance by having PC, SEBS, and Geniomer fibers bend onto a load cell using the integrated tendon‐driven mechanism, resulting in different blocking forces (Figure 1d). We selected an experimental configuration whereby the force sensor was placed orthogonally to the fiber at its initial state with a bending angle of 0°. Upon actuation, the bending path was blocked by the force sensor at a bending angle of ≈10°. The force sensor data revealed a maximum force of 66.3 mN for PC fibers, while 2.7 mN and 1.3 mN were exerted by the SEBS and Geniomer fibers, respectively. The difference in output force by an order of magnitude between the thermoplastic PC and the two thermoplastic elastomers SEBS and Geniomer, results from the significantly larger elastic modulus of PC. The same trend is observed for SEBS and Geniomer, where the stiffer SEBS fibers generate higher bending forces. The output forces recorded here are lower compared to previously reported macroscopic pressure‐ and tendon‐driven actuators^[^ ^46^, ^47^, ^48^ ^]^ due to the significantly smaller dimensions of the fibers (diameter of ≈1.5 mm). Another force‐limiting factor is the friction between the tendons and the supporting lumens, which is a limitation of tendon‐driven actuators.^[^ ^49^, ^50^ ^]^ In this work, friction is diminished through the application of ethanol within the lumens as a lubricant. Nonetheless, as shown below, the output forces are sufficient to accomplish various multi‐functional tasks.

Ultimately, we select SEBS as the primary material for our soft robotic fibers, because it exhibits a favorable rheological behavior (a high loss modulus that dominates over a low storage modulus for a considerable temperature range), and a relatively low surface tension, yielding high structural integrity during processing. Moreover, its mechanical behavior (a high elongation at break and an intermediate stiffness) leads to high compliance and intermediate force outputs. Note that both PC and Geniomer are alternative materials that can be employed in applications where a higher output force or higher mechanical compliance, respectively, is required. Based on this materials and fabrication platform we explored a variety of different fiber designs (Figure 1e). We also demonstrate the integration of multiple functional elements through the feeding mechanism, such as optical guides or metallic wires, which result in intricate soft‐hard material constructs with small diameters and extended lengths. These examples highlight the level of structural complexity that can be achieved, such as channels with diameters of ≈100 µm and inter‐channel spacings of 10s of µm. All fiber designs integrate three lumens that each hosts a tendon consisting of a nylon line or metallic wire with diameters of 70–200 µm. To assure free translation of the tendons within the lumens at minimum friction, we introduce ethanol as a lubricant. Note that the ethanol does not cause any consequential swelling of SEBS (Figure S1, Supporting Information). We qualitatively demonstrate the range of bending angles and directions using a fiber of diameter 1.5 mm and length 80 mm (Figure 1f). Moreover, we illustrate the adaptability of the fibers, as well as their mechanical compatibility with the human body, arising from the softness and deformability of SEBS, by having a robotic fiber of diameter 700 µm safely wrap around a finger (Figure 1g).

To effectively control the soft robotic fibers, we designed a peripheral control unit. Its hardware encompasses three servo motors, each pulling by controlled amounts on one of the tendons, a microcontroller and computer, as well as, function‐specific components, such as photoemitters and detectors. The complementary custom software allows intuitive fiber control by a user through keyboard direction buttons, as well as the launching of path scripts or autonomous control protocols (for details of the setup, refer to Note S1 and Figure S2, Supporting Information).

**Figure 2:**
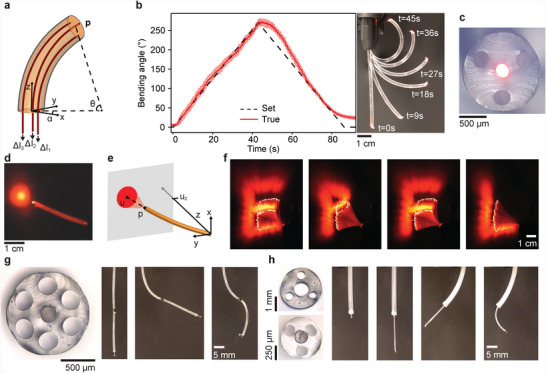
Soft robotic fibers motion control. a) Schematic of the fiber configuration controlled by tendon displacements. b) Bending angle ramp, shown as a comparison of the set and true bending angle, as well as, a merged sequence of photographs. c) Optical micrograph of the cross‐section of a fiber integrating a light guide that emits red light. d) Photograph of the fiber emitting light onto a screen. e) Schematic of the light projection. f) Merged sequence of photographs of predefined motion patterns executed by the fiber. g) An alternative fiber design for high motion complexity, shown by an optical micrograph of the cross‐section and a sequence of photographs of different configurations. h) Another strategy of high‐degree‐of‐freedom motion, achieved by inserting a thinner robotic fiber within a larger one.

At the heart of the control system lies our kinematic model, formulated upon the constant‐curvature approximation. With this framework, the high dimensionality of the soft robot is reduced to a 3D configuration space, parameterized by the fiber length *l*, bending angle *θ*, and direction angle *α*, or task space defined by the coordinates of the end effector **p** (**Figure** **2**a). The details of the model are outlined in Note S2 and Figure S3, Supporting Information. We first validate our inverse kinematics in a 1D experiment, where a bending angle sequence at constant bending direction is input into the system. In parallel, we capture the true bending angle with a camera and computer vision. The soft robotic fiber follows the set bending angle ramp closely (Figure 2b) and satisfies the constant‐curvature approximation (Figure S4, Supporting Information). Note that in this experiment, we limited the bending angle to a maximum of 270°, which we deem beyond the requirements of most applications, although our robotic fibers can bend to significantly larger extents, as high as 540° (Figure S5, Supporting Information).

Next, we validate our controls in two dimensions by varying the bending direction in addition to the bending angle. Addressing the core of our innovation, we employ a first multifunctional soft robotic fiber design equipped with an optical guide at its core (Figure 2c) and aim to deliver light to spatially defined areas. Light from a source connected at the proximal end is emitted out of the distal end onto a screen (Figure 2d). Thus, the inverse kinematics are extended by an additional level consisting of the coordinates of the center of the light spot **u** (Figure 2e). In the experiment, we compile a sequence of **u** coordinates into a script similar to a G‐code, which is commonly employed for the controls of computer numerical control machines or 3D printers. Upon input of the list, the robotic fiber automatically executes the trajectories, successfully tracing the shape of predefined letters (Figure 2f). The shown images are created by superimposing the frames of a video of the experiment by areas of highest intensity (Movie S1, Supporting Information).

In a concluding remark regarding motion control, we present two strategies for increasing the degrees of freedom, currently limited to bending about two axes in the basic fiber design. In the first approach, the cross‐sectional structure is altered thanks to the design freedom of our process, to include six rather than three lumens, allowing two sets of three tendons to be distributed in the structure (Figure 2g). By terminating the first set of tendons halfway along the length and the second at the fiber end, the fiber is essentially separated into two segments that can each be separately actuated. In the second approach, a thinner basic robotic fiber is introduced within a larger robotic fiber featuring a large lumen at its center (Figure 2h). In addition to decoupled bending of both fibers, the thinner fiber can be advanced and retracted within the larger fiber, adding another translational degree of freedom. These examples illustrate the complexity of motion that can be achieved as well as the modularity of our soft robotic fibers.

**Figure 3:**
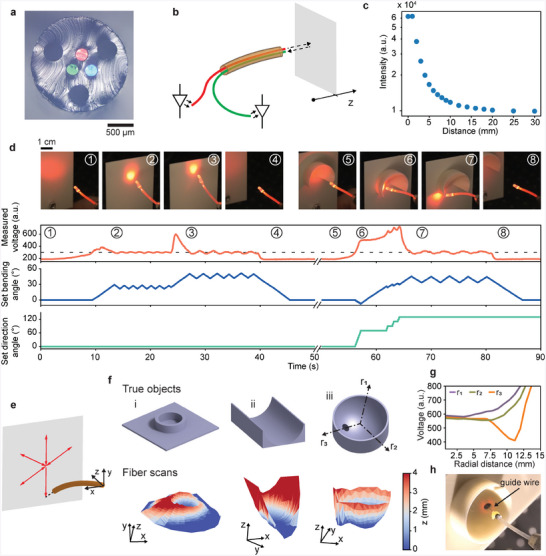
Proximity sensing with soft robotic fibers. a) Optical micrograph of the cross‐section of a fiber integrating three optical guides, that are each emitting a differently colored light. b) Schematic of the working principle of the proximity sensing. c) Intensity of received light as a function of distance to the reflecting obstacle. d) Autonomous obstacle avoidance experiment, shown by a sequence of photographs, as well as, a plot of the measured photodarlington transistor voltage and set bending angle and direction as function of experiment time. The dashed line indicates the threshold value for initiating the bending. The encircled numbers relate the photographs to the corresponding times in the plot. e) Schematic of the fiber motion pattern to scan over the surface of an unknown object. f) Comparison between the true objects, shown as 3D models, and the reconstructions obtained with the fiber. g) Measured proximity signals for three exemplary radial scan orientations as indicated on object iii in panel (f). h) Photograph of the fiber facing the hole in the object, a configuration which was autonomously executed based on collected proximity signals. A guidewire is manually advanced through one of the fiber lumens into the targeted hole.

To further highlight the opportunities of our approach, we address the perception of the surroundings as another important capability in robots, particularly in medical settings where the environments are fragile and dynamic. We seek to accomplish exteroception^[^ ^23^, ^51^, ^52^ ^]^ in our soft robotic fibers through optical displacement sensing^[^ ^53^, ^54^ ^]^ with several fiber‐embedded optical guides (**Figure** **3**a). The sensor configuration consists of one guide connected to a light source and another to a photodetector at the proximal end, acting as transmitting and receiving lines (Figure 3b). The intensity of light reflected at an obstructing surface depends sensitively on the distance and decreases monotonically beyond a peak at ≈0.5 mm (Figure 3c). In a spectroscopic analysis (Figure S6, Supporting Information), we find that distances can be quantified at submillimeter resolution up to 18 mm at a wavelength of 650 nm. Thus, we employ a light‐emitting diode with peak wavelength 645 nm and a photodarlington transistor in subsequent experiments.

The performance of fiber optic displacement sensors is known to be influenced by several factors, including the environmental light, the design of the probe, the reflectivity and relative angle of the reflecting surface, and the refractive index of the surrounding medium.^[^ ^53^ ^]^ The tests were completed in a darkroom to reduce the effect of environmental light. High measurement sensitivity is typically achieved by using low‐loss optical guides and high‐reflecting surfaces. We employ industrial‐grade optical guides made of PMMA with losses of 150–300 dB km^−1^ in the robotic fibers because of their superior mechanical properties compared to stiff and brittle silica optical guides. Additionally, we use white surfaces in the proximity tests rather than mirrors, to replicate conditions expected from targeted applications. Moreover, the white surfaces act as diffuse reflecting surfaces, which minimize the effect of the variable orientation of the probe to the surface on the measurement. Finally, to quantify the effect of the refractive index of the surrounding medium, we tested the sensor in deionized water and water with an absorbing agent (Figure S7, Supporting Information). Besides a shift of the intensity–distance curve, which can be resolved by recalibration upon operation in a medium of different refractive index, the sensor performance remains unchanged.

We introduce the measure of proximity as an exteroceptive feedback to close the control loop, setting the stage for autonomous movement and obstacle avoidance. In a first experiment, we implement an on–off algorithm whereby the bending angle is increased when a threshold value of reflected intensity is exceeded due to near‐impact proximity of an obstacle, and reduced again when the condition is not met. As shown Figure 3d, stages 1–4, the soft robotic fiber adjusts its configuration according to the continuous measure of reflected intensity, successfully avoiding contact to an approaching object, as well as, returning to its initial position when the disturbance is removed. In a second test, we increase the complexity of the task by introducing an obstacle that cannot be avoided through 1D bending. We take advantage of the omnidirectional bending capability of our robotic fibers and extend the algorithm by an additional condition: When an increase in bending angle causes a decrease in distance rather than the expected increase, the step is reversed threefold and the bending direction is altered. Also in this case, the robotic fiber could autonomously find a configuration to avoid an impact with the approaching obstacle (Figure 3d, stages 5–8). The complete experiment is shown in Movie S2, Supporting Information.

Next, we show that the soft robotic fibers can also act as imaging devices, where the proximity signals are used in conjunction with the known fiber configurations to reconstruct the 3D spatial surroundings (for details, refer to Note S3 and Figure S8, Supporting Information). To test our 3D mapping concept, we implement a radial scanning protocol (Figure 3e) that is automatically executed on three objects with defined geometries (Figure S9, Supporting Information). As shown in Figure 3f, the computed representations based on the fiber scans match the true shapes. The imaging capability is expected to be particularly beneficial in applications where the thin robotic fibers are employed in areas that are inaccessible to larger diagnostic tools, such as endoscopes equipped with a camera. Moreover, particular areas of interest in the surroundings are readily identified and can be used as reference points for autonomous task completion. We demonstrate this capability with the cup‐shaped object, which features an orifice. The intensity profile obtained for the path crossing over the hole exhibits a distinctive minimum, which is absent for the two other exemplary orientations (Figure 3g). Once the scan is completed, the robotic fiber automatically snaps to the position of minimum intensity, as it was instructed to do before the experiment. The resulting fiber configuration provides an ideal access for subsequent navigation and manipulation, which we illustrate by manually advancing a guidewire of diameter 150 µm through one of the lumens of the fiber directly into the targeted opening (Figure 3h and Movie S3, Supporting Information).

**Figure 4:**
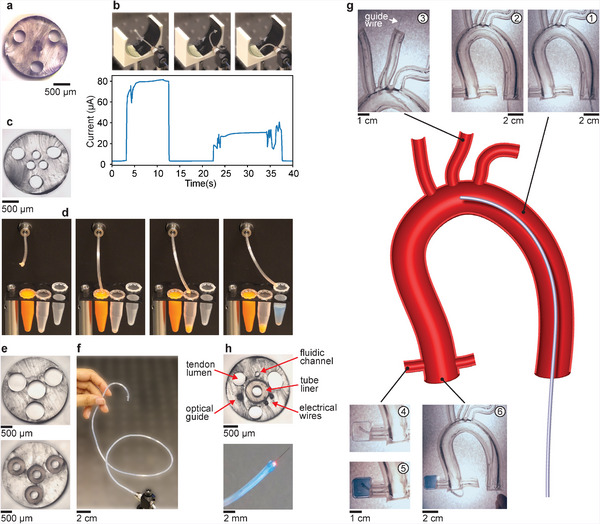
Electrical and fluidic functionalities, and advanced navigation with soft robotic fibers. a) Optical micrograph of the cross‐section of a fiber integrating three metallic wires. b) Sequence of photographs and measured current during an electrical probing experiment. c) Optical micrograph of the cross‐section of a fiber integrating three microchannels at its center. d) Sequence of photographs of a fluidic experiment. e) Optical micrographs of the cross‐sections of a fiber integrating four PTFE liners (bottom) and of the same fiber with the liners removed. f) Photograph of a 65 cm‐long robotic fiber with a steerable soft tip and a stiff body incorporating the PTFE liners. g) Model of the aortic arch and sequence of photographs of a robotic fiber navigating the vessels, placing a guidewire, and delivering a fluid. h) Optical micrograph of the cross‐section (top) and photograph (bottom) of a fiber integrating 3 tendon‐dedicated lumens, a PTFE liner, an optical guide, two electrical wires, and a fluidic channel.

In addition to optical guides, the soft robotic fibers can also host metallic wires to electrically stimulate and record spatially distributed targets, as demonstrated by a fiber integrating three wires of diameter 125 µm (**Figure** **4**a). The wires are connected to an electrometer on the proximal end and left exposed at the distal end of the fiber. Thus, if the fiber tip comes in contact with a conducting surface, the circuit between two of the three wires is closed and samples can be electrically characterized through current measurements. To test this functionality, we control the fiber via direction keys of the computer keyboard and aim at electrically probing two different conductive hydrogels with conductivities, 1.1 and 0.3 mS m^−1^. The fiber readily bends from the initial configuration toward the desired points on the samples, where sufficient force is applied to create a stable electrical contact. Indeed, the measured current corresponds to the conductivities of the samples (Figure 4b and Movie S4, Supporting Information).

Next, we incorporate several channels in the soft robotic fibers (Figure 4c). Through the use of pumps connected at the proximal end, the channels enable the targeted delivery or suction of fluids. In the experiment, we rely once again on the keyboard controls to move the robotic fiber and accurately address several spatially distributed vials (Figure 4d and Movie S5, Supporting Information). In a first step, we use one of the channels to suck fluid out of one of the vials and deliver it to the vial next to it. Subsequently, with one of the other channels, we fill a third vial with a separate fluid to highlight the multiplexing capability.

Finally, as an exemplary application highlighting the potential of our soft robotic fibers, we consider minimally invasive procedures, where long tubular structures are advanced within body cavities and the tip is actuated to steer the device in the desired direction. We incorporate four stiff polytetrafluoroethylene (PTFE) liners within the soft thermoplastic elastomer cladding during the thermal drawing step (Figure 4e), three of which host the tendons and the fourth centered liner can serve an additional purpose. By mechanically removing the liners at the distal end in post‐processing, the resulting continuous fiber of diameter 2.25 mm comprises a soft steerable tip of length 5 cm and a stiff pushable body of length 60 cm (Figure 4f). The disparate length and diameter result in an aspect ratio of the soft robot of 290, but aspect ratios as high as 1150 were also achieved (Figure S10, Supporting Information). We demonstrate the capability of the device by navigating the fiber within a model of the aortic arch and carrying out a set of tasks (Figure 4g and Movie S6, Supporting Information). In a first step, the fiber is manually advanced and the tip is actuated by keyboard controls to conform to the curvature of the model (Stages 1 and 2). Next, the tip is steered in the opposite direction, allowing the fiber to enter the targeted vessel, where a guide wire is placed by advancing it through the center liner of the fiber (Stage 3). Wires within catheters are typically employed for navigation purposes but can also carry mechanical tools, such as stents, to the targeted area. Subsequently, the fiber is maneuvered to another vessel and a fluid is injected through the center liner and delivered precisely to the desired location (Stages 4 and 5), showcasing the administration of a contrast agent to the coronary artery. Finally, the 3D motion of the robotic fiber is demonstrated at the far end of the model (Stage 6) and the fiber is retracted.

## 3 Conclusion

We have shown that the thermal drawing of microstructured fibers sets the stage for soft robotic devices with extreme aspect ratios and multiple advanced functionalities. We presented several material options for the fibers, including a thermoplastic and two thermoplastic elastomers, which all exhibit the rheological processing attributes for thermal drawing but varying mechanical properties, resulting in adaptable actuation performance. Additionally, we explored 11 different cross‐sectional designs featuring outer diameters as small as 700 µm and internal features sizes of 10s of µm, as well as aspect ratios as high as 10^3^. Complex and accurate motion is achieved by a tendon‐driven mechanism complemented by a kinematic model and additional modalities are evoked by embedded light guides, electrical wires, fluidic channels, or PTFE liners—all of which can be combined in a single fiber (Figure 4h). The soft robotic fibers are particularly promising in minimally invasive procedures, where they can perceive, image, and autonomously adapt according to their spatial environments using optical displacement sensing, as well as, probe electric properties and deliver fluids and tools to spatially distributed targets.

## 4 Experimental Section

### Material Characterization

The three cladding materials of the fibers, polycarbonate (PC, Polymaker), SEBS (Kraton), and Geniomer, a copolymer of aliphatic isocyanite and polydimethylsiloxane (Wacker) were characterized in terms of its rheological and mechanical properties. The rheology of disks of diameter 25 mm and thickness 1 mm, fabricated by compression molding (custom press, Lauffer), was analyzed by oscillatory shear rheology (AR 2000, TA Instruments), where the temperature was increased from 50 to 200 °C, under oscillatory shear strain of frequency 1 Hz and amplitude 1%. The mechanical properties were quantified under standard tensile testing (Z005 with 50 and 500 N load cell, Zwick/Roell), using fiber samples of diameter 1.5 mm and clamped length 25 mm, and at a speed of 50 mm min^‐1^. The swelling experiment was performed by introducing a weighted sampled of SEBS into a sealed container containing ethanol. In regular intervals, the sample was removed, dried, and weighted to track the amount of absorbed ethanol.

### Soft Robotic Fiber Fabrication and Preparation

The fabrication of the preform differed for the thermoplastic elastomers (SEBS, Geniomer) compared to the thermoplastic (PC). For the thermoplastic elastomers, granules of the cladding material, were filled into custom steel molds, which featured the desired outer shape as well as interior mold cores. The granules were compression‐molded at ≈0.5 bar, 180 °C for 12 h. For PC, the preform was 3‐dimensionally printed. Next, the preforms were thermally drawn into fibers using a custom draw tower at a set middle‐zone‐temperature of 200 °C for SEBS, 240 °C for Geniomer, and 250 °C for PC and at a feed speed of 1 mm min^−1^ and take‐up speeds ranging from 100 to 900 mm min^−1^, resulting in diameter scale‐down ratios of 10 to 30. For fibers featuring additional functional elements such as optical guides, a feeding mechanism was employed as an extension to the thermal drawing process. Before starting the process, the 1D elements were introduced in designated holes within the preform. The significant decrease in size of the cross‐section during the process, led to the fed elements being caught within the narrowing holes by the flowing cladding material and being continuously unspooled. Fed elements included tendons (bend‐and‐stay multipurpose 304 stainless steel wires, super‐elastic nitinol wire, or clear nylon line, McMaster‐Carr), optical guides (optical grade plastic optical fiber unjacketed, Edmund Optics), electric wires (bend‐and‐stay multipurpose 304 stainless steel wires, super‐elastic nitinol wire, McMaster‐Carr), and PTFE liner tubing (high‐temperature tube sleeving, McMaster‐Carr). In fiber post‐processing, the fibers were first cut into suitable segment lengths. The tendons and other functional elements were exposed at the ends through a solvent (toluene, Sigma‐Aldrich) or mechanical treatment. On one end, the tendons were fixed by tying knots or applying epoxy glue. On the other end, the tendons and other functional elements were interfaced with the peripheral control unit (see Note S1, Supporting Information). For the fiber integrating four PTFE liners, the liners were removed from a small section of the fiber by pulling on them on the other end, resulting in a long continuous fiber with a soft tip without the liners and a stiff body integrating the liners. Finally, for all fiber samples a drop of ethanol was applied on the tip of the fiber, where it was sucked into the lumens of the tendons by capillary effect, acting as a lubricant during actuation. Excess ethanol was removed with the corner of a tissue paper. The fabricated fibers were assessed using optical microscopy (DM 2700 M, Leica).

### Force Sensing

The blocking force generated by PC, SEBS, and Geniomer thermally drawn fibers with dimensions of 5 cm length and ≈1.5 mm diameter was compared. The experimental setup consisted of 500 g load cell made of a straight aluminum bar (TAL221, SparkFun) equipped with a platform onto which the robotic fibers pressed. The original height to the force sensor from the fibers’ distal end tip was set to 10 mm, while the bending angle varied from 0° to 10°, at which point the trajectory was blocked by the platform. The data was collected using an amplifier (HX711, SparkFun) coupled to a microcontroller (Arduino Uno, Arduino).

### Motion Characterization

The soft robotic fibers were fixed and actuated with the peripheral control unit (see Note S1, Supporting Information). For the 1D bending, a camera (C270, Logitech) was orientated perpendicularly to the bending direction of the fiber. During the bending angle ramp, the time, set bending angle, and camera images were continuously recorded. In post‐processing, the video feed was analyzed using computer vision (OpenCV in Python), where the coordinates of the two fiber extremities were extracted, yielding the fiber's true bending angle. To characterize the motion in two dimensions, a soft robotic fiber with a light guide at its center was employed, where the light guide was connected to a red light‐emitting diode (IF E96E, Industrial Fiber Optics) at the proximal end. Light emitted out of the distal end of the light guide was projected onto a screen. The experiment, consisting of executing planed trajectories, was recorded by a camera (RX100, Canon). The cumulative light path was visualized in post‐processing using frame blending by maximum intensity in video editing software (Premiere Pro, Adobe).

### Proximity Sensor Characterization

A soft robotic fiber featuring three light guides at its center was used for proximity sensing experiments. All experiments were conducted in a darkroom to reduce the effect of environmental light. In the spectroscopic analysis, one light guide was connected to a white light source (HL‐2000, Ocean Optics) and another light guide to a photodetector (USB2000+VIS‐NIR‐ES, Ocean Optics). A white object with a flat surface (3D printed object with polylactic acid (Prusament PLA Vanilla White, Prusa Research)) orthogonal to the fiber was displaced in a controlled fashion and the spectrum of reflected light recorded at every increment at a constant integration time of 1000 ms. Due to the rough surface of the printed object, it was considered a diffuse reflecting surface. In subsequent experiments, a practical light source (IF E96E, Industrial Fiber Optics) and photodetector (IF D93, Industrial Fiber Optics) were employed. For testing different media, the fiber tip was submersed in a beaker, which was displaced in a direction parallel to the fiber using a translation stage. A white PTFE sheet (GoodFellow) placed in the beaker served as the reflecting surface (Figure S7, Supporting Information). In the obstacle avoidance test, 3‐dimensionally printed objects of white PLA were displaced relative to the fiber and the measured intensity signal and set fiber configuration were continuously recorded. For the fiber scanning experiment, 3‐dimensionally printed objects (Figure S9, Supporting Information) were kept at a constant distance while the fiber moved atop them. The scan involved a bending angle step size of 2° and range of 2°–34° and a bending direction step size of 10° and range of 0°–360°, resulting in a total 612 points that can be used for the reconstruction of the shape (for more details see Note S3, Supporting Information). For the experiment involving the guide wire, a fiber was used where one of the light guides was mechanically extracted and replaced by a stiff metallic wire (spring‐back multipurpose 304 stainless steel wire, McMaster‐Carr). Once the scan procedure was completed and the fiber automatically moved to the desired position angled at the targeted hole, the steel wire was manually moved forward, penetrating the hole.

### Electrical Probing Test

The electrical probing experiment was performed using a soft robotic fiber featuring three metallic electrodes at its center (super‐elastic nitinol wire, McMaster‐Carr). The wires were mechanically exposed at both extremities. On the proximal end, the wires were interfaced to an electrometer (HF2LI lock‐in amplifier, Zurich Instruments), where two wires were connected conjointly at the first pole and the third wire at the second pole. The current was continuously recorded under an electrical stimulation of frequency 100 kHz and amplitude 1 V, while the fiber motion was conducted using the keyboard control. The tested samples consisted of conductive hydrogels composed of gelatine‐glycerol, where the conductivity was tuned by a controlled amount of polypyrrole resulting in two types of samples with admittivities 1.1 and 0.3 mS m^−1^.

### Microfluidic Channels Test

The fluidic experiment was performed using a soft robotic fiber featuring three channels at the center. On the proximal end, the two of the channels were connected by inserting syringe tips, leading through tubing to syringes. Water with differently colored dyes was used as an illustrative fluid. The fiber motion was conducted using the keyboard control, and fluid suction and delivery was achieved by manually operating the syringes.

### Navigation of the Model of the Aortic Arch

The model of the aortic arch was fabricated using molding of silicone (Sylgard, 184 Silicone Elastomer) in custom 3D printed molds. The navigation experiment was conducted using a 65‐cm‐long soft robotic fiber featuring four PTFE liners. The liners were removed in a 5‐cm‐long section at one fiber end, resulting in a 5‐cm‐long soft tip. Three nylon lines were placed in the three outer liners, fixed on the distal end, and linked to the peripheral control unit on the proximal end. Additionally, a syringe tip was inserted into the center liner. In the experiment, the robotic fiber was manually advanced within the model and the tip steered using the keyboard controls of the control unit. Navigation of the fiber to the targeted vessels was achieved by advancing and retracting the fiber as well as actuating the tip. At the desired location, a nitinol wire was manually advanced and subsequently retracted through the center liner. Ethanol with a blue dye was administered to the desired location by injecting it using a syringe into the center liner of the fiber.

## Conflict of Interest

The authors declare no conflict of interest.

## Supporting information

Supporting Information

Supplemental Movie 1

Supplemental Movie 2

Supplemental Movie 3

Supplemental Movie 4

Supplemental Movie 5

Supplemental Movie 6

## Data Availability

The data that support the findings of this study are available from the corresponding author upon reasonable request.

## References

[1] D. Rus , M. T. Tolley , Nature 2015, 521, 467. 26017446 10.1038/nature14543

[2] F. Renda , M. Giorelli , M. Calisti , M. Cianchetti , C. Laschi , IEEE Trans. Rob. 2014, 30, 1109.

[3] J. Paek , I. Cho , J. Kim , Sci. Rep. 2015, 5, 10768. 26066664 10.1038/srep10768 PMC4463937

[4] L. Lindenroth , C. Duriez , J. Back , K. Rhode , H. Liu , in 2017 IEEE/RSJ Int. Conf. Intell. Robot. Syst, IEEE, Vancouver, BC, Canada 2017, p. 2923.

[5] J. Z. Gul , Y. J. Yang , K. Y. Su , K. H. Choi , Soft Rob. 2017, 4, 224. 10.1089/soro.2016.0042 29182084

[6] X. Ji , X. Liu , V. Cacucciolo , M. Imboden , Y. Civet , A. El Haitami , S. Cantin , Y. Perriard , H. Shea , Sci. Rob. 2019, 4, eaaz6451. 10.1126/scirobotics.aaz6451 33137720

[7] Y. Kim , G. A. Parada , S. Liu , X. Zhao , Sci. Rob. 2019, 4, eaax7329. 10.1126/scirobotics.aax7329 33137788

[8] W. Dou , G. Zhong , J. Cao , Z. Shi , B. Peng , L. Jiang , Adv. Mater. Technol. 2021, 6, 2100018.

[9] C. Laschi , B. Mazzolai , M. Cianchetti , Sci. Rob. 2016, 1, eaah3690. 10.1126/scirobotics.aah3690 33157856

[10] L. Hines , K. Petersen , G. Z. Lum , M. Sitti , Adv. Mater. 2017, 29, 1603483. 10.1002/adma.201603483 28032926

[11] M. Cianchetti , C. Laschi , A. Menciassi , P. Dario , Nat. Rev. Mater. 2018, 3, 143.

[12] T. G. Thuruthel , B. Shih , C. Laschi , M. T. Tolley , Sci. Rob. 2019, 4, eaav1488. 10.1126/scirobotics.aav1488 33137762

[13] R. L. Truby , L. Chin , A. Zhang , D. Rus , Sci. Adv. 2022, 8, eabq4385. 35947669 10.1126/sciadv.abq4385 PMC9365281

[14] R. V. Martinez , J. L. Branch , C. R. Fish , L. Jin , R. F. Shepherd , R. M. D. Nunes , Z. Suo , G. M. Whitesides , Adv. Mater. 2013, 25, 205. 22961655 10.1002/adma.201203002

[15] Y. Muramatsu , T. Kobayashi , S. Konishi , 2015 28th IEEE International Conference on Micro Electro Mechanical Systems (MEMS), Estoril, Portugal 2015, pp. 166–167.

[16] V. Kashyap , A. Caprio , T. Doshi , S.‐J. Jang , C. F. Liu , B. Mosadegh , S. Dunham , Sci. Adv. 2020, 6, eabc6800. 33188028 10.1126/sciadv.abc6800 PMC7673747

[17] S. Fusco , H.‐W. Huang , K. E. Peyer , C. Peters , M. Häberli , A. Ulbers , A. Spyrogianni , E. Pellicer , J. Sort , S. E. Pratsinis , B. J. Nelson , M. S. Sakar , S. Pané , ACS Appl. Mater. Interfaces 2015, 7, 6803. 25751020 10.1021/acsami.5b00181

[18] X. Hu , A. Chen , Y. Luo , C. Zhang , E. Zhang , Comput. Assisted Surg. 2018, 23, 21. 10.1080/24699322.2018.1526972 30497292

[19] J. Burgner‐Kahrs , D. C. Rucker , H. Choset , IEEE Trans. Rob. 2015, 31, 1261.

[20] M. Runciman , A. Darzi , G. P. Mylonas , Soft Rob. 2019, 6, 423. 10.1089/soro.2018.0136 PMC6690729 30920355

[21] G.‐B. Jin , M.‐J. Wang , D.‐Y. Zhao , H.‐Q. Tian , Y.‐F. Jin , J. Mater. Process. Technol. 2014, 214, 50.

[22] S. Cho , E. Lee , S. Jo , G. M. Kim , W. Kim , Polymers 2020, 12, 1628. 32707865 10.3390/polym12081628 PMC7464286

[23] A. Leber , B. Cholst , J. Sandt , N. Vogel , M. Kolle , Adv. Funct. Mater. 2018, 29, 1802629.

[24] B. Gorissen , M. De Volder , D. Reynaerts , Biomed. Microdevices 2018, 20, 73. 30105633 10.1007/s10544-018-0317-1

[25] T. Gopesh , J. H. Wen , D. Santiago‐Dieppa , B. Yan , J. S. Pannell , A. Khalessi , A. Norbash , J. Friend , Sci. Rob. 2021, 6, eabf0601. 10.1126/scirobotics.abf0601 PMC9809155 34408094

[26] T. J. Jones , E. Jambon‐Puillet , J. Marthelot , P.‐T. Brun , Nature 2021, 599, 229. 34759362 10.1038/s41586-021-04029-6

[27] D. Fan , X. Yuan , W. Wu , R. Zhu , X. Yang , Y. Liao , Y. Ma , C. Xiao , C. Chen , C. Liu , H. Wang , P. Qin , Nat. Commun. 2022, 13, 5083. 36038593 10.1038/s41467-022-32859-z PMC9424246

[28] K. P. Becker , Y. Chen , R. J. Wood , Adv. Funct. Mater. 2020, 30, 1908919.

[29] K. Ikuta , H. Ichikawa , K. Suzuki , D. Yajima , Proc. IEEE Int. Conf. Rob. Autom. 2006, 2006, 4161.

[30] N. Tan , X. Gu , H. Ren , Mech. Mach. Theory 2018, 122, 197.

[31] A. Stilli , H. A. Wurdemann , K. Althoefer , 2014 IEEE/RSJ International Conference on Intelligent Robots and Systems, Chicago, IL, USA 2014, pp.2476–2481.

[32] M. A. Schmidt , A. Argyros , F. Sorin , Adv. Opt. Mater. 2016, 4, 13.

[33] W. Yan , A. Page , T. Nguyen‐Dang , Y. Qu , F. Sordo , L. Wei , F. Sorin , Adv. Mater. 2019, 31, 1802348. 10.1002/adma.201802348 30272829

[34] M. Bayindir , A. F. Abouraddy , F. Sorin , J. D. Joannopoulos , Y. Fink , Opt. Photonics News 2004, 15, 24.

[35] M. E. M. K. Abdelaziz , L. Tian , T. Lottner , S. Reiss , K. During , G.‐Z. Yang , M. Bock , B. Temelkuran , in 2021 ISMRM & SMRT Annual Meeting & Exhibition, 15–20 May 2021, https://www.ismrm.org/21/program-files/O-74.htm.

[36] Y. Guo , S. Jiang , B. J. B. Grena , I. F. Kimbrough , E. G. Thompson , Y. Fink , H. Sontheimer , T. Yoshinobu , X. Jia , ACS Nano 2017, 11, 6574. 28570813 10.1021/acsnano.6b07550

[37] S. Shadman , T. Nguyen‐Dang , T. D. Gupta , A. G. Page , I. Richard , A. Leber , J. Ruza , G. Krishnamani , F. Sorin , Adv. Funct. Mater. 2020, 30, 1910283.

[38] Y. Qu , T. Nguyen‐Dang , A. G. Page , W. Yan , T. D. Gupta , G. M. Rotaru , R. M. Rossi , V. D. Favrod , N. Bartolomei , F. Sorin , Adv. Mater. 2018, 30, 1707251. 10.1002/adma.201707251 29799143

[39] A. Leber , A. G. Page , D. Yan , Y. Qu , S. Shadman , P. Reis , F. Sorin , Adv. Funct. Mater. 2020, 30, 1904274.

[40] A. Leber , C. Dong , R. Chandran , T. D. Gupta , N. Bartolomei , F. Sorin , Nat. Electron. 2020, 3, 316.

[41] C. Dong , A. Leber , T. D. Gupta , R. Chandran , M. Volpi , Y. Qu , T. Nguyen‐Dang , N. Bartolomei , W. Yan , F. Sorin , Nat. Commun. 2020, 11, 3537. 32669555 10.1038/s41467-020-17345-8 PMC7363815

[42] M. Rein , V. D. Favrod , C. Hou , T. Khudiyev , A. Stolyarov , J. Cox , C.‐C. Chung , C. Chhav , M. Ellis , J. Joannopoulos , Y. Fink , Nature 2018, 560, 214. 30089921 10.1038/s41586-018-0390-x

[43] T. Nguyen‐Dang , A. C. de Luca , W. Yan , Y. Qu , A. G. Page , M. Volpi , T. D. Gupta , S. P. Lacour , F. Sorin , Adv. Funct. Mater. 2017, 27, 1605935.

[44] T. N. Dang , I. Richard , E. Goy , F. Sordo , F. Sorin , J. Appl. Phys. 2019, 125, 175301.

[45] Z. Xin , B. Du , S. Yan , S. Du , J. Ding , Z. Yang , W. Ren , J. Biomater. Sci., Polym. Ed. 2014, 25, 1045. 24854325 10.1080/09205063.2014.918458

[46] K. C. Galloway , P. Polygerinos , C. J. Walsh , R. J. Wood , in 2013 16th International Conference on Advanced Robotics (ICAR), Montevideo, Uruguay, IEEE, Manhattan, New York, US 2013, pp. 1–6.

[47] Y. Li , Y. Chen , T. Ren , Y. Li , S. H. Choi , Soft Rob. 2018, 5, 567. 10.1089/soro.2017.0090 29924683

[48] J.‐H. Lee , Y. S. Chung , H. Rodrigue , Sci. Rep. 2019, 9, 11251. 31375746 10.1038/s41598-019-47794-1 PMC6677814

[49] G. Subramani , M. R. Zinn , 2015 IEEE International Conference on Robotics and Automation (ICRA), Seattle, WA, USA, IEEE, Manhattan, New York, US 2015, 610–617.

[50] J. Jung , R. S. Penning , M. R. Zinn , Adv. Robot. 2014, 28, 557.

[51] H. Zhao , K. O'Brien , S. Li , R. F. Shepherd , Sci. Rob. 2016, 1, eaai7529.

[52] P. A. Xu , A. K. Mishra , H. Bai , C. A. Aubin , L. Zullo , R. F. Shepherd , Sci. Rob. 2019, 4, eaaw6304. 10.1126/scirobotics.aaw6304 PMC6853625 31723716

[53] H. Z. Yang , X. G. Qiao , D. Luo , K. S. Lim , W. Chong , S. W. Harun , Measurement 2014, 48, 333.

[54] H. A. Rahman , A. I. C. Ani , S. W. Harun , M. Yasin , R. Apsari , H. Ahmad , J. Biomed. Opt. 2012, 17, 071308. 22894469 10.1117/1.JBO.17.7.071308

