# Supplementary material for: Highly Integrated Multi‐Material Fibers for Soft Robotics
Source: Adv Sci (Weinh). 2022 Nov 22;10(2):2204016. doi: 10.1002/advs.202204016 (PMC9839840; doi:10.1002/advs.202204016)
Supplement: Supplementary file 1 — Supporting Information [file ADVS-10-2204016-s006.pdf]

ADVANCED SCIENCE
Open Access

Supporting Information

for *Adv. Sci.*, DOI 10.1002/advs.202204016

Highly Integrated Multi-Material Fibers for Soft Robotics

*Andreas Leber, Chaoqun Dong, Stella Laperrousaz, Hritwick Banerjee, Mohamed E. M. K. Abdelaziz, Nicola Bartolomei, Bastien Schyrr, Burak Temelkuran and Fabien Sorin**

Supporting Information

Highly integrated multi-material fibers for soft robotics

*Andreas Leber, Chaoqun Dong, Stella Laperrousaz, Hritwick Banerjee, Mohamed E.M.K. Abdelaziz, Nicola Bartolomei, Bastien Schyrr, Burak Temelkuran & Fabien Sorin**

Supporting Note 1: Peripheral control unit

Actuation of the soft robotic fibers is driven by three embedded tendons. To accurately control the bending angle and bending direction of the fibers, the three tendons are fixed at the distal end of the fibers and pulled by specific amounts. This translation as well as the interfacing with the diverse additional embedded functionalities are accomplished with the peripheral control unit. Its design can be separated into hardware and software. Both parts are shown in Figure S3.

On the hardware side, the proximal end of the robotic fiber is clamped in a dedicated vise. From the vise, the tendons are led into a distributor element, which contains three Teflon-coated channels, one for each tendon. The purpose of the distributor is to spatially separate the tendons, which are very close in the thin fibers and thus difficult to actuate individually. From the distributor, each tendon is fixed to the arm of a servo motor. The servo motors are fixed on rails and can thus be translated, allowing the tension of the tendons at the zero position to be controlled. The three servos are interfaced to a microcontroller, which allows the angle to be precisely set. The commands are sent to the microcontroller via a PC. Besides controlled translation of the tendons, the control unit also serves as an interface to additional functionalities in the fibers. For optical functionality, photoemitters and detectors are included, which are connected to the optical guides within the robotic fibers. The control unit includes three differently colored LEDs (red, green, blue) as well as a photodarlington transistor, each embedded into suitable electronic circuits and continuously addressed by the microcontroller. For electronic functionalities, the unit also features a wire terminal, into which individual thin wires can be inserted. The lines from the terminal can be connected to a dedicated electrometer for electrical characterization. Finally, fluidic functionality is supported by syringe tips that can be inserted in the microchannels of the fiber at the proximal end. On the other side, the syringe tips are connected to a syringe for manual injection and suction or a peristaltic pump for automized fluidic tasks. To analyze experiments in the aftermath, a camera is also connected to the control unit.

The actuation and additional functionalities are coordinated by the PC, which runs a custom graphical user interface. The program is written in Python 3 and includes the modules Numpy, Matplotlib, Serial, Tkinter, and OpenCV for multi-dimensional array operation, real-time plotting and visualization, communication with the microcontroller, providing a graphical user interface, and recording images and videos, respectively. The actuation controls are eased by the implementation of a kinematic model, which is discussed in Supporting Note 2. The movement of the robotic fiber can be instructed using different ways:

(i) The absolute arm angles for each servo can directly be input. (ii) In addition to unique values, each servo can also be instructed to execute a servo angle ramp, where starting and end position as well as step size and step time are set. (iii) The desired bending angle and bending direction of the robotic fiber can be input, which are automatically converted to servo angle instructions. (iv) With the keyboard direction buttons the fiber can be intuitively moved in the desired direction, based on cartesian or polar coordinate systems according to user preference. (v) Previously planned trajectories can also be executed. The trajectories are input as sequences of bending angle and bending direction in the form of a text file. Upon initiation, the program consecutively runs through the coordinates in the sequence, automatically enacting the planed trajectory. (vi) The fiber can also be controlled without specific user input. In this case, the proximity sensor signal is used as a feedback in a closed-loop controller. Depending on the implemented controller, such as on-off control using a threshold value, the fiber autonomously adjusts its configuration based on the measured signal.

Regardless of the chosen actuation control, the graphical user interface displays essential information of the system to the user in real-time. This includes a bar chart of the absolute servo arm angles, a 3-dimensional visualization of the current fiber configuration, and a bar chart of collected signals, such as photodarlington transistor voltage in the proximity sensing device. Finally, the software also features the possibility of saving all collected data, including time, set servo angles, set fiber configurations, measured signals, and a synchronized video stream from the camera.

Supporting Note 2: Kinematic model

The controls of the soft robotic fibers are underlain by a kinematic model which provides a sequential mapping of the different operating spaces. The model is presented in its inverse form, as in most application we aim at calculating the inputs to achieve a set end effector position.

The first mapping consists of relating the task space, parameterized by the end effector coordinates **p** = (p_x, p_y, p_z), to the configuration space defined by the parameters fiber length l, bending angle θ, and bending direction α, as defined in Figure S3A:[1]

$$\alpha = arctan\left(\frac{p_y}{p_x}\right) \tag{2.1}$$

$$\theta = arccos\left(\frac{1-\frac{{p_y}^2}{{p_z}^2 \cdot sin^2(\alpha)}}{\frac{{p_y}^2}{{p_z}^2 \cdot sin^2(\alpha)}+1}\right) \tag{2.2}$$

The second mapping relates the configuration space to the joint space, parameterized by the displacements of the three tendons Δl_i with i=1,2,3. As a robot-dependent mapping, the structural parameters eccentricity e and tendon angle φ_i, shown in Figure S3B, must be included:[2]

$$\Delta l_i = e \cdot \theta \cdot cos(\alpha - \varphi_i) \tag{2.3}$$

In practical experiments, we extended this ideal case by two terms. First, we included an additional term to avoid tendon slacking without influencing bending angle and direction, which causes a compression of the fiber and thus a slight shortening of length, but does not alter the bending angle or bending direction. Second, we added a correction factor f_i for each tendon to account for slightly different zero positions during setup. The correction factor was obtained through a one-angle visual calibration, typically at a bending angle of 90°.

$$\Delta l_i = f_i \cdot (e \cdot \theta \cdot cos(\alpha - \varphi_i) + e \cdot \theta) \tag{2.4}$$

The third and final mapping connects the joint space to the actuator space, which depends on the setup geometrical parameters servo arm length a_i and servo distance d_i, and yields the servo arm angle β_i, shown in Figure S3C:

$$\beta_i = arccos\left(\frac{{a_i}^2 + {d_i}^2 - (l_{i0} + \Delta l_i)^2}{2 \cdot a_i \cdot d_i}\right) \qquad (2.4)$$

where l_{i0} is the initial length of the tendon segment between the distributor element and the point on the servo arm where the tendon is fixed. It depends on the initial servo arm angle setting:

$$l_{i0} = \sqrt{{a_i}^2 + {d_i}^2 - \cos\beta \cdot 2 \cdot a_i \cdot d_i} \tag{2.4}$$

With these three mappings, the coordinates of the end effector are converted to the direct inputs of the peripheral control units in the form of the arm angles of the three servos.

Supporting Note 3: Fiber scan 3-dimensional shape reconstruction

The soft robotic fibers developed in this work can act as imaging devices, where the proximity signals are used in conjunction with the known fiber configurations to reconstruct the 3-dimensional spatial surroundings. First, the proximity sensor must be calibrated. This is done by recording the photodarlington transistor voltages for specific distances of a reflecting white surface to the tip of the robotic fiber. An example of a calibration curve is shown in Figure S9A.

We find that the relationship between the measured voltage V and the distance d can be best described using a function of the form:

$$d = a_0 + \frac{a_1}{V} + \frac{a_2}{V^2} + \frac{a_3}{V^3} \tag{3.1}$$

The parameters a_i, i=0,1,2,3 are found by fitting the function to the calibration data. Based on this calibration curve, a distance can be directly calculated from a measured photodarlington transistor voltage signal.

Next, we must determine the kinematic relationship between, on one hand, the coordinates **u** of the targeted point on the reflecting surface, and, on the other hand, the measured distance and set fiber configuration. We first derive in 2 dimensions using the schematic shown in Figure S9B:

$$\boldsymbol{u} = \begin{pmatrix} \frac{l}{\theta} \cdot (1 - \cos\theta) + \sin\theta \cdot d \\ 0 \\ \frac{l}{\theta} \cdot \sin\theta + \cos\theta \cdot d \end{pmatrix} \tag{3.2}$$

We extend the expression to 3 dimensions by applying the rotation matrix around the z-axis by the bending direction angle:

$$\boldsymbol{u} = \begin{pmatrix} \cos\alpha & -\sin\alpha & 0 \\ \sin\alpha & \cos\alpha & 0 \\ 0 & 0 & 1 \end{pmatrix} \cdot \begin{pmatrix} \frac{l}{\theta} \cdot (1 - \cos\theta) + \sin\theta \cdot d \\ 0 \\ \frac{l}{\theta} \cdot \sin\theta + \cos\theta \cdot d \end{pmatrix} \tag{3.3}$$

$$
= \begin{pmatrix} \cos\alpha \cdot \left(\frac{l}{\theta} \cdot (1-\cos\theta) + \sin\theta \cdot d\right) \\ \sin\alpha \cdot \left(\frac{l}{\theta} \cdot (1-\cos\theta) + \sin\theta \cdot d\right) \\ \frac{l}{\theta} \cdot \sin\theta + \cos\theta \cdot d \end{pmatrix}
$$

Using this expression, every set of configuration angles and distance, which is determined using the intensity of reflected light and the calibration curve, yields the coordinates of a point on the surface. Finally, all the points from the scan are plotted, resulting in the 3-dimensional reconstruction.

References Supporting Information

[1] F. Qi, F. Ju, D. Bai, Y. Wang, B. Chen, *Int. J. Med. Robot. Comput. Assist. Surg.* **2019**, *15*, DOI 10.1002/rcs.2007.

[2] M. M. Dalvand, S. Nahavandi, R. D. Howe, *IEEE Trans. Robot.* **2018**, *34*, 1215.

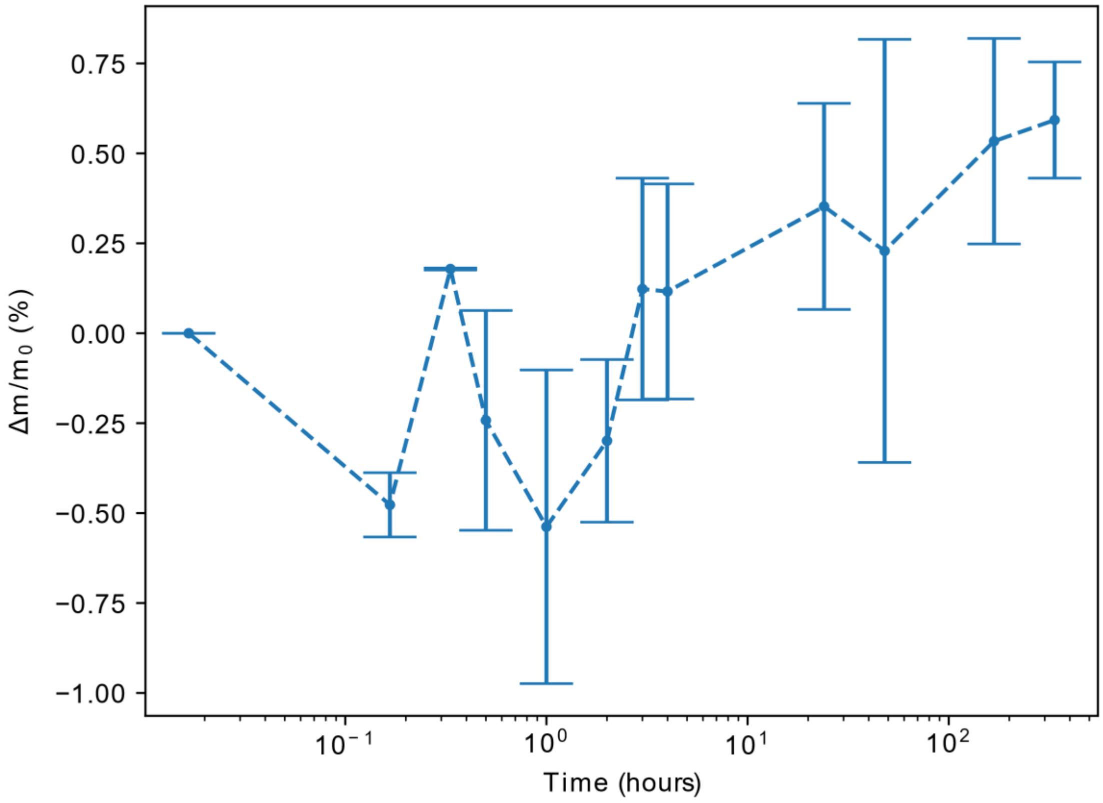

Figure S1.
Swelling of SEBS samples of dimensions 0.7mm×2cm×2cm in ethanol. This experiment was

repeated on 3 different samples to assess the mean value of the swelling ratio, along with its standard deviation.

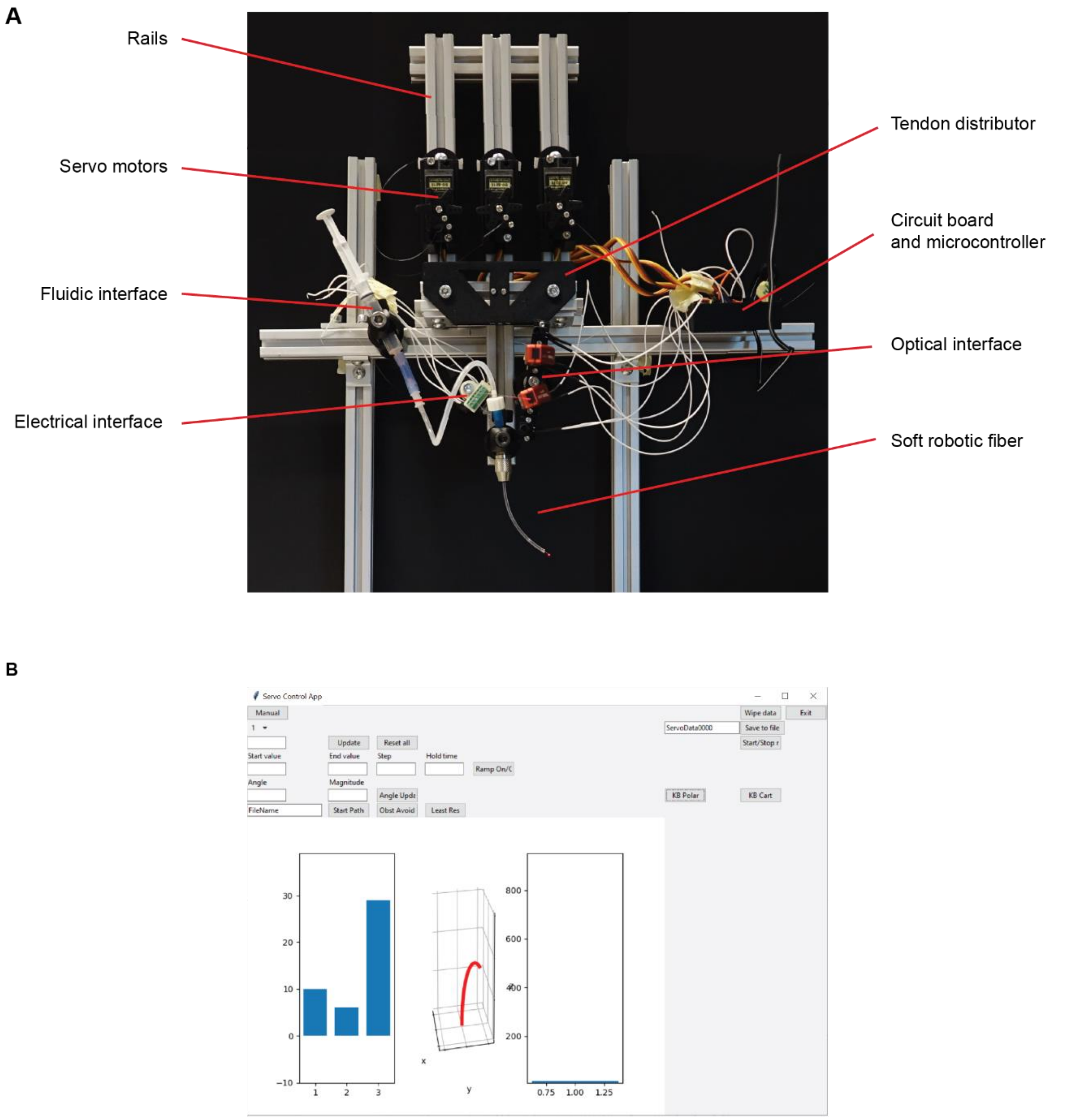

Figure S2.

Peripheral control unit. a) Experimental setup to control and interface with the soft robotic fibers. b) Screenshot of the graphical user interface.

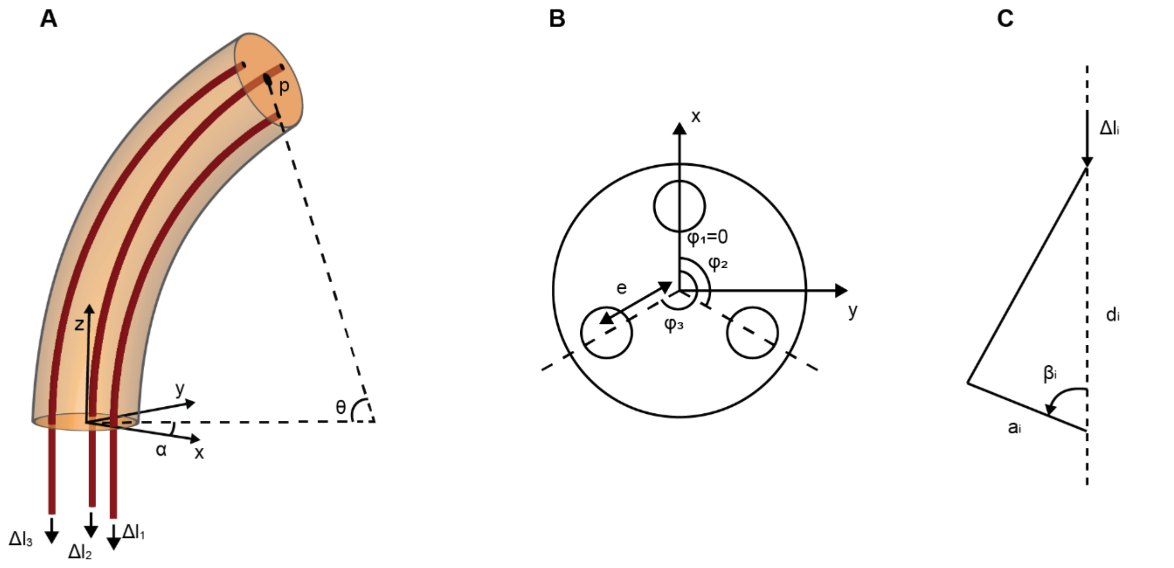

Figure S3.
Parameters of the kinematic model. a) Schematic outlining the 3-dimensional bending of the fiber. The end effector coordinates **p** depend on the bending angle θ and bending direction α. The configuration of the fiber is set by controlled displacements of the tendons Δl_i at the proximal end. b) Schematic of the fiber cross-section. How much each tendon must be pulled depends on the structural parameters of the fiber, including the eccentricity e. The bending direction α is indicated to emphasize that the tendon displacements depend on their placement in the fiber relative to the bending direction. c) Schematic to outline the relationship between tendon displacement and set servo arm angle βi through the setup parameters arm length a_i and servo distance from tendon distributor d_i.

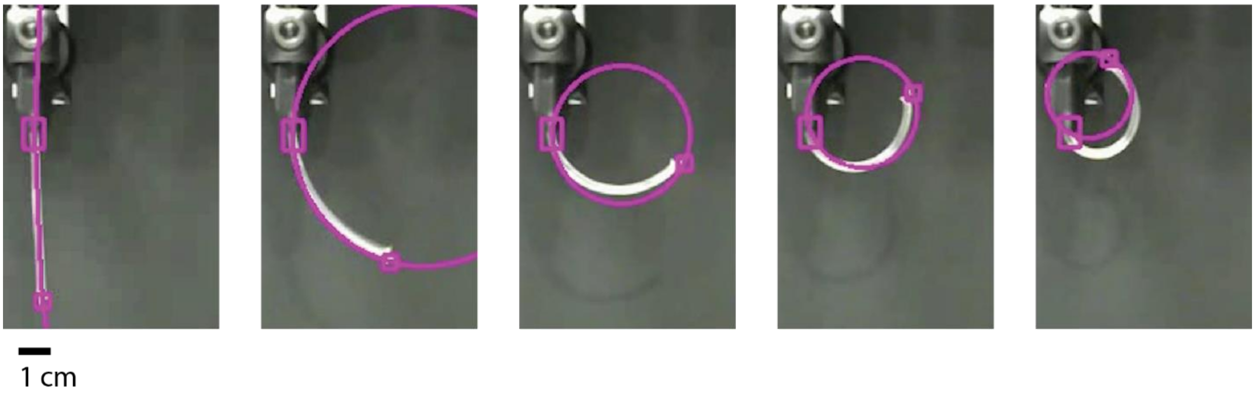

Figure S4.
1-dimensional bending angle ramp. Sequence of images as the bending angle is continuously increased. The two extremities of the soft robotic fiber are selected as objects that are tracked throughout the experimental video. A circle is fitted based on the coordinates of the two points. As shown, it corresponds to the curvature of the fiber.

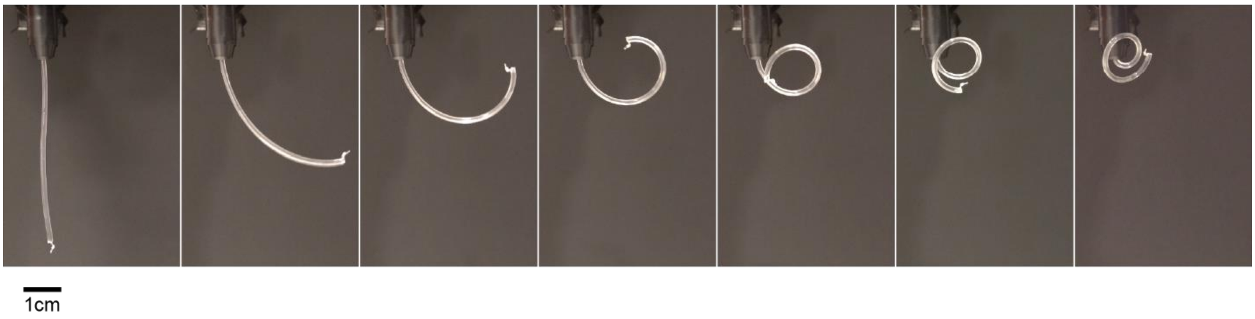

Figure S5.
Curvature ramp with a maximum bending angle of 540°.

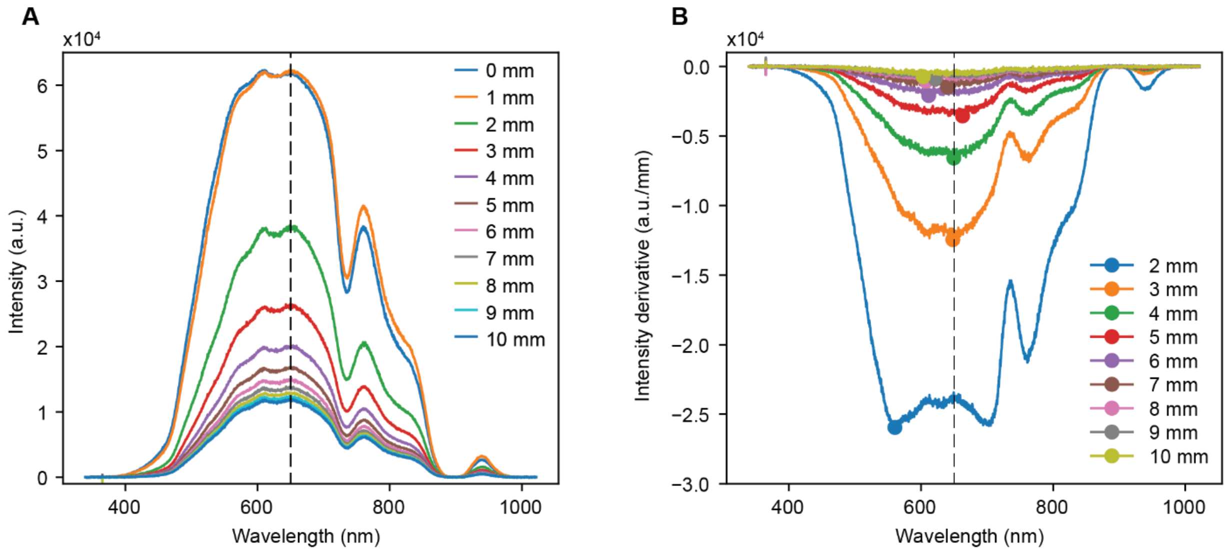

Figure S6.
Spectroscopic analysis of proximity sensor. Light from a white light source is transmitted through one of the light guides, reflected at a white surface, and collected through a second light guide to a spectrometer. a) Spectrum of reflected light for different distances of the fiber tip to the reflecting surface. The wavelength 650 nm is indicated as a dashed line. b) Spectrum of derivative of intensity with respect to distance for different distances. The negative peak for each curve is indicated by a solid marker. The wavelength 650 nm is highlighted as a dashed line.

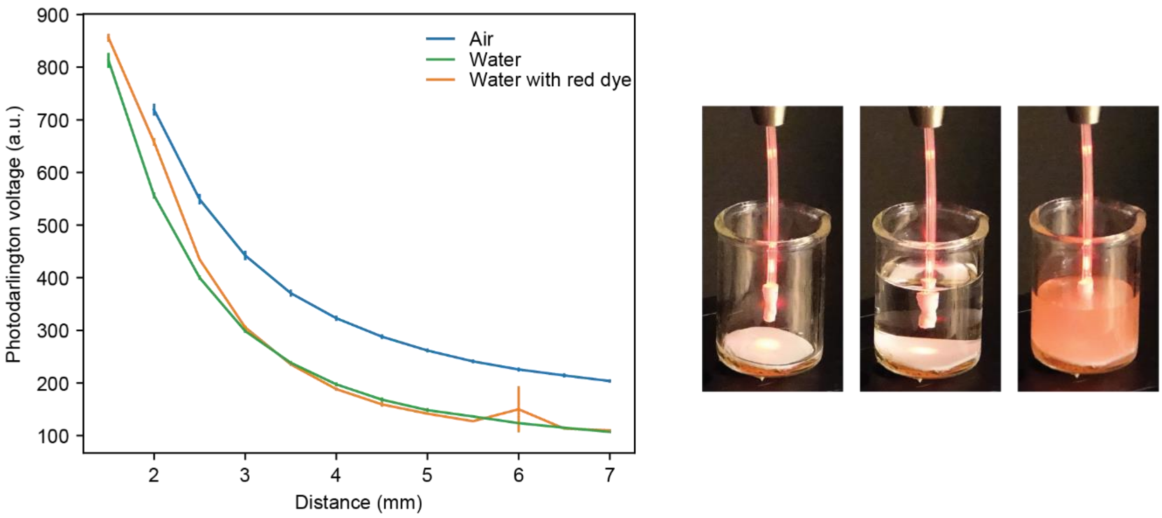

Figure S7.
Proximity sensor performance in different media. A reflective surface is translated respective to the fiber tip in different media. The shown voltage of the photodarlington transistor corresponds to the reflected light intensity from a red LED.

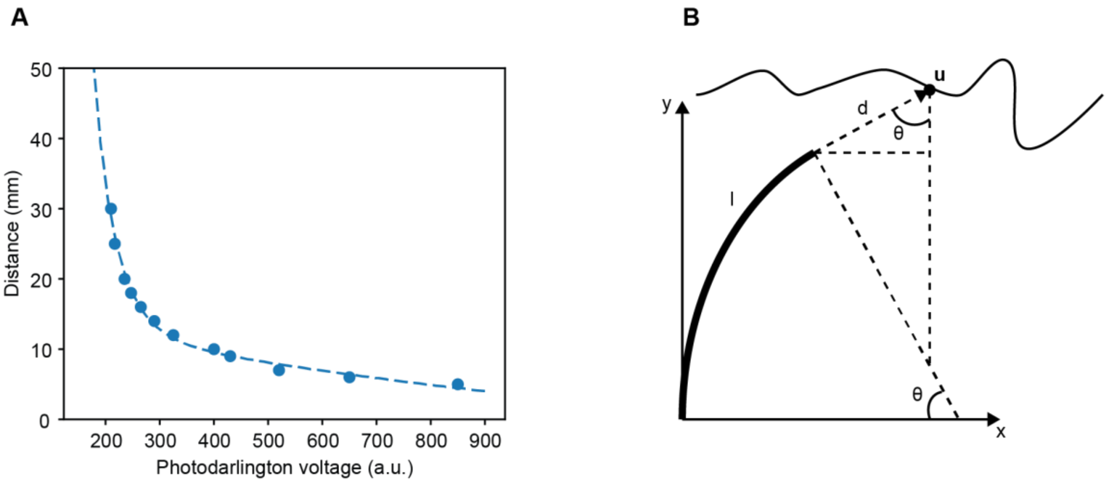

Figure S8.
Reconstruction of 3-dimensional surroundings using proximity sensor. a) Calibration curve obtained by fitting a function to the data obtained from a calibration experiment. b) Schematic outlining the relevant geometrical parameters to calculate the coordinates **u** on the surface of an object from the measured distance d and fiber bending angle θ.

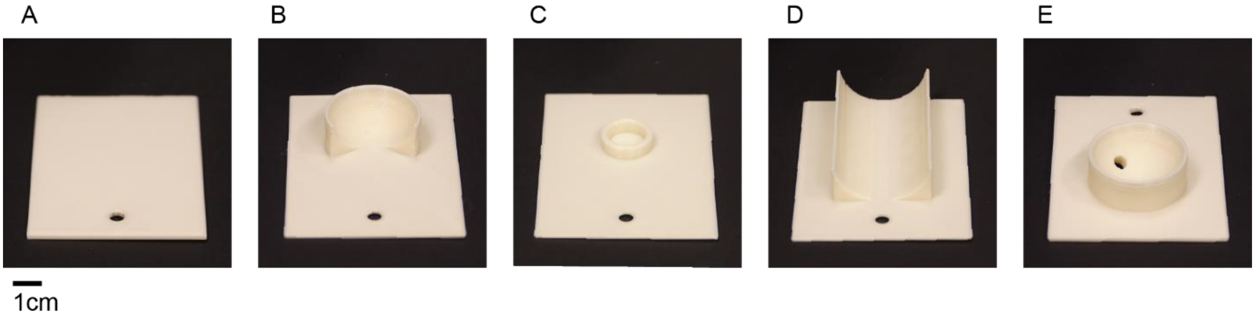

Figure S9.
Photographs of all 3-dimensionally printed objects employed in the experiments of the proximity sensor. a) Employed for obstacle avoidance test stages 1-4. b) Employed for obstacle avoidance test stages 5-8. c) ,d) , e) Employed for fiber scans.

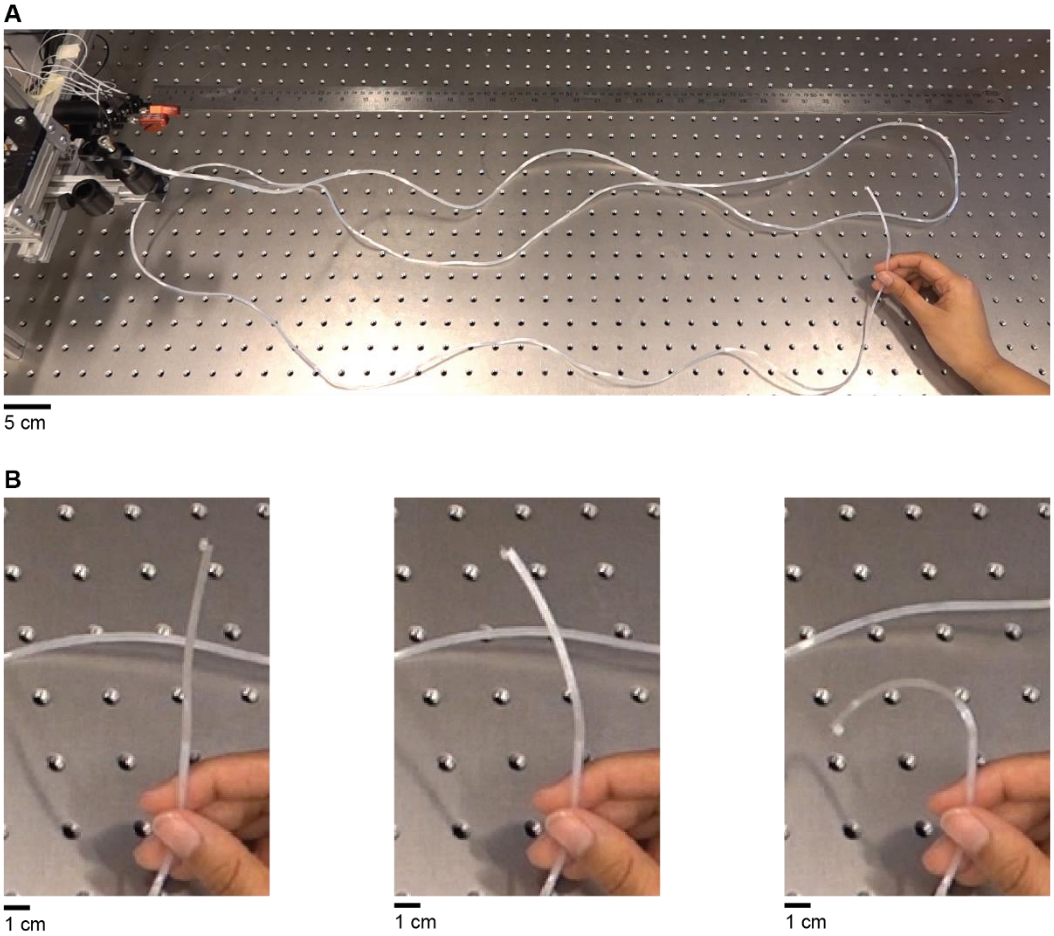

Figure S10.
Long soft robotic fiber. a) Photograph of a long robotic fiber with a soft steerable tip and a stiff body integrating PTFE liners. The length is 3.17 m and the diameter 2.75 mm, resulting in an aspect ratio of the device of 1150. b) Sequence of photographs of the steerable tip of the fiber to illustrate the actuation.

Movie S1.
Two-dimensional control of a soft robotic fiber integrating an optical guide at its core. Red light is emitted at the distal fiber end onto a screen. A list of coordinates is introduced in the control unit, which is translated to the servo motor inputs, resulting in automatic execution of trajectories. The left image shows the actual video captured from the experiment. The right image is edited in post-processing by stacking the video frames by intensity, to illustrate the complete path traced on the screen by the light emitted from the fiber.

Movie S2.
Autonomous obstacle avoidance of the soft robotic fibers, enabled by optical displacement sensing. In the first experiment, a flat screen is approached to the distal end of the fiber, resulting in an increase of reflected light intensity. When an arbitrarily defined threshold of the measured signal is reached, the fiber bends to avoid collision. When the disturbance is removed, the fiber returns to its original configuration. In the second experiment, a complex shape is approached that cannot be avoided by linear bending. The fiber also alters its bending direction angle to avoid the obstacle.

Movie S3.
Autonomous identification and execution of the ideal fiber configuration to access an orifice. The soft robotic fiber scans its environment in a radial pattern, collection distance measures through optical displacement sensing. A distinct intensity minimum is registered when the fiber crosses over an orifice in the object. Upon completion of the scan, the fiber autonomously snaps to the position over the hole. A metallic wire is manually advanced through a lumen in the fiber directly into the targeted hole.

Movie S4.
Electrical probing with a soft robotic fiber, integrating three metallic wires at its core. Two gels with high and low electrical conductivity are placed within the vicinity of the fiber. Through keyboard controls the fiber is instructed to contact consecutively the two different gels. The current between two of the wires is measured simultaneously, show-casing the ability to electrical characterize samples with the fibers.

Movie S5.
Removal and delivery of multiple fluids using a soft robotic fiber integrating three microchannels at its core. Through keyboard controls, the fiber is instructed to pump liquid out of a first vial and deliver it to a second adjacent vial. Additionally, a different liquid is delivered to a third vial.

Movie S6.
Demonstration of 65 cm-long soft robotic fiber with a steerable tip for the navigation and the completion of tasks within a replica of the aortic arch. The individual steps in the procedure are described in the captions of the video.
